# Exostosin 1 regulates cancer cell stemness in doxorubicin-resistant breast cancer cells

**DOI:** 10.18632/oncotarget.19737

**Published:** 2017-07-31

**Authors:** Sarala Manandhar, Chang-Gu Kim, Sun-Hee Lee, Soo Hyun Kang, Nikita Basnet, You Mie Lee

**Affiliations:** ^1^ BK21 Plus Multi-Omics Based Creative Drug Research Training Team (22A20154413076), National Basic Research Laboratory of Vascular Homeostasis Regulation, Research Institute of Pharmaceutical Sciences, College of Pharmacy, Kyungpook National University, Daegu 41566, South Korea

**Keywords:** breast cancer, cancer stem cell, exostosin1, ALDH^+^, CD44^+^/CD24^-^

## Abstract

Cancer stem cells (CSCs) are associated with cancer recurrence following radio/chemotherapy owing to their high resistance to therapeutic intervention. In this study, we investigated the role of exostoxin 1 (EXT1), an endoplasmic reticulum (ER)-residing type II transmembrane glycoprotein, in cancer cell stemness. DNA microarray analysis revealed that doxorubicin-resistant MCF7/ADR cells have high levels of EXT1 expression compared to its parental cell line, MCF7. These cells showed significantly higher populations of CSCs and larger populations of aldehyde dehydrogenase (ALDH^+^) and CD44^+^/CD24^-^cells, as compared to MCF7 cells. siRNA-mediated knockdown of EXT1 in MCF7/ADR cells significantly reduced cancer stem cell markers, populations of ALDH^+^and CD44^+^/CD24^-^ cells, mRNA and protein expression for CD44, and mammosphere number. Furthermore, epithelial mesenchymal transition (EMT) markers and migratory behavior were also repressed with reduced EXT1. In an *in vitro* soft agar colony formation assay, EXT1 knockdown by short hairpin RNA (shRNA) reduced the colony formation ability of these cells. Based on these results, we suggest that EXT1 could be a promising novel target to overcome cancer cell stemness in anthracycline-based therapeutic resistance.

## INTRODUCTION

Understanding the mechanism of drug resistance is one of the key challenges in the fight against cancer. Existing genetic alteration in cancer cells before therapy can confer therapeutic failure in conventional therapy [1]. Overexpression of certain genes has been reported to be involved in *de novo* chemoresistance and recurrence of cancer. In addition, mutation or overexpression of certain drug targets, overexpression of ABC transporters, increased anti-apoptotic machinery and damage repair, and enhanced drug inactivation mechanism are involved in the intrinsic or acquired resistance to chemotherapy [2–4].

Cancer stem cells (CSCs) or tumor initiating cells (TICs) are a small group of cells within tumors that can self-renew, initiate cancer, and further maintain and differentiate to generate cellular heterogeneity in tumors [5–8]. The concept of CSCs was originally coined by Lapidot and colleagues in hematologic cancer [9]; later, the functional role of individual CSCs in the formation of tumors was experimentally and clinically evidenced [10]. CSCs were initially identified in human cortical glial tumors on the basis of cell surface markers [11]. Subsequently, CSCs were more precisely identified and characterized in various human tumors. Based on large number of reports, CSCs in solid tumors are primarily identified by cell surface markers such as CD24-, CD44+, CD133+, aldehyde dehydrogenase (ALDH^+^) activity, and Hoechst efflux [5, 12–15]. CSCs are associated with resistance to radio/chemotherapy, and therefore believed to be associated with recurrence of more aggressive cancer [16–18]. Furthermore, chemoresistant cancer cells are enriched with CSCs [19, 20], and chemotherapy can also increase subpopulations of cells with CSC-like properties [21]. In addition, epithelial mesenchymal transition (EMT) inducers can induce breast cancer cells to breast CSCs enriched with the CD44^+^/CD24^-^ configuration [22, 23]. Similarly, acquisition of paclitaxel resistance in epithelial ovarian carcinoma (EOC) promotes EMT-like behavior [24] and chemotherapy treatment can enhance EMT markers in breast cancer [25, 26], revealing that the emergence of CSCs occurs as a result of EMT, to an extent [27].

Based on the status of the hormonal receptor, breast tumors are classified as estrogen receptor positive (ER+) and-negative (ER–) [28]. Patients with ER+ tumors are frequently treated with hormonal therapies and/or with chemotherapy to weaken estrogen responses. Doxorubicin hydrochloride (Adriamycin, Rubex) is one among several commonly used chemotherapeutic agents in the treatment of breast cancer. However, many reports suggest that the antitumor effect of doxorubicin (doxo) induces cell death by apoptosis or through cell cycle arrest [29, 30], it can also exhibit its antiproliferative effect through impairment of estrogen stimulated growth and survival responses [31]. Furthermore, several clinical studies have reported that ER+ breast cancer patients are less responsive to chemotherapy than their ER- counterparts [31]. Similar to this observation, ER+ breast cancer cell lines have also validated the presence of physiologic estrogen levels, disrupting the effects of chemotherapy in *in vitro* studies [32, 33]. With this knowledge, we grasped the importance of understanding the mechanism of drug resistance and attempted to investigate the underlying molecular signature of chemotherapeutic resistance to enhance the effectiveness of chemotherapy.

Exostoxin 1 (EXT1) is an endoplasmic reticulum (ER)-residing type II transmembrane glycoprotein that is involved in the biosynthesis of cell surface heparan sulfate (HS) [34, 35]. However, mutations in EXT1 are known to be the cause of hereditary multiple exostoses (HME), an autosomal dominant disorder characterized by benign bone tumors on the active bone growth areas [36], emphasizing its role as a tumor suppressor, increased EXT1 DNA copy number alteration (DCNA) has also been reported in aggressive bone tumor [37]. In addition, Khoontawad *et al.* has shown increased expression of EXT1 in plasma of human cholangiocarcinoma (CCA) bile duct cancer [38]. Furthermore, HS chains are reported to be crucial for the growth and survival of multiple myeloma (MM) cells and *in vivo* knockdown of EXT1 was verified for the suppression of its growth [39], implicating the possible role of EXT1 in cancer progression. However, to date, there is no evidence that EXT1 regulates CSC properties, and critical analyses and characterization are needed to understand its role in carcinogenesis. Here, we endeavored to investigate the role of EXT1 in cancer cell stemness using the doxo-resistant human breast cancer cell line, MCF7/ADR, established by exposing MCF7, a doxo-sensitive human breast cancer cell line, to serially escalated doses of doxo up to 1 μM. We observed overexpressed EXT1 in MCF7/ADR cells can facilitate and regulate cancer cell stemness, including increased ALDH^+^and CD44^+^/CD24^-^ population, and growth of larger and more numerous mammospheres in MCF7/ADR cells. Furthermore, knockdown of EXT1 not only repressed cancer cell stemness but also downregulated EMT markers and abolished cell surface HS, suggesting EXT1 as a potential molecular target to overcome cancer cell stemness in doxo-mediated therapeutic resistance.

## RESULTS

### Doxorubicin resistance enhances CSC properties in MCF7 cells

**Figure 1:**
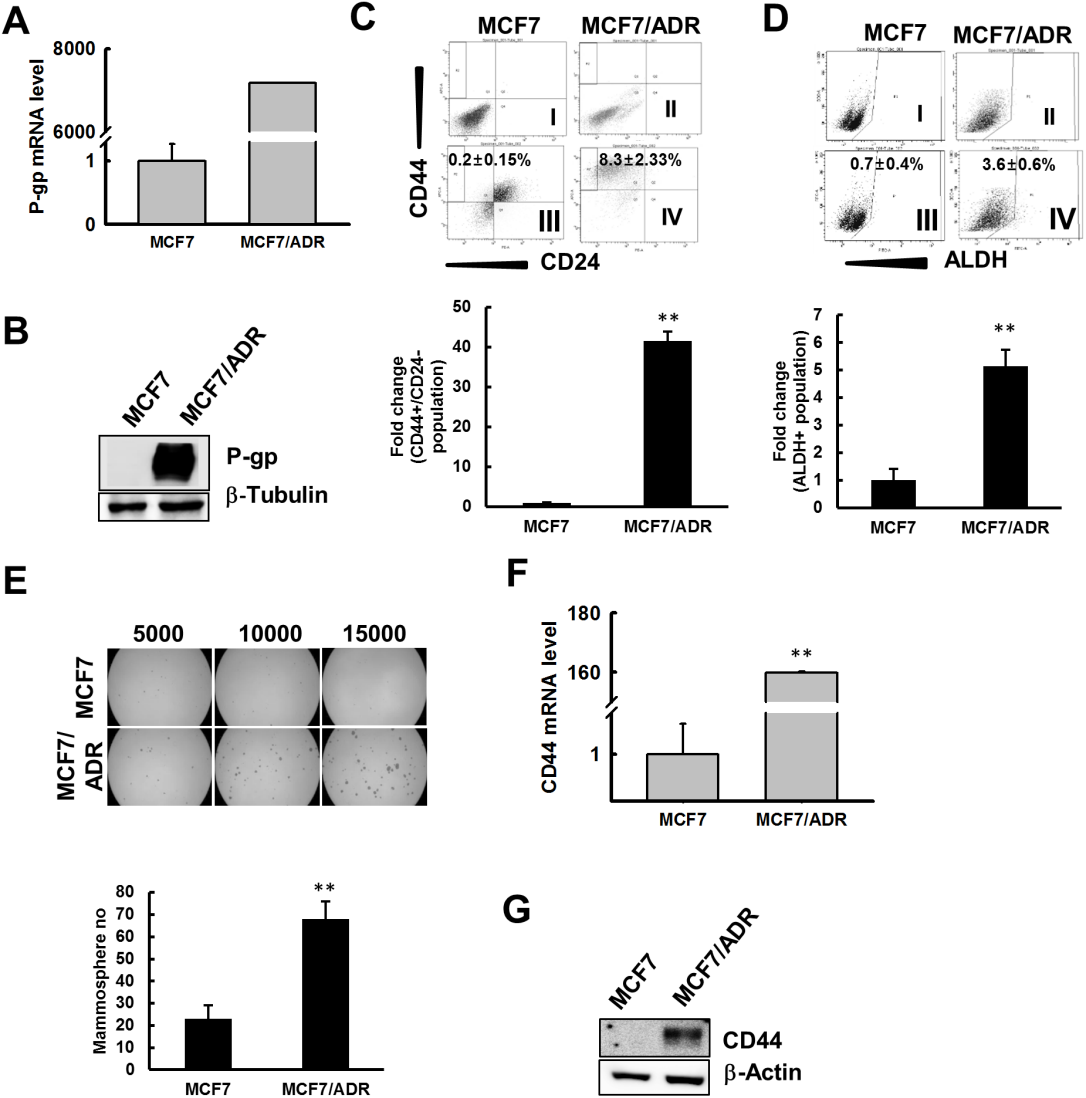
MCF7/ADR cells enriched for CSC characteristics **(A)** Total mRNA was extracted from MCF7 and MCF7/ADR cells and qRT-PCR was performed to detect P-gp. Fold change in MCF7/ADR versus MCF7 was determined and graphed. **(B)** P-gp protein level determined by immunoblot analysis. β-tubulin was used as an internal control to normalize the expression of target protein. Fold change in MCF7/ADR versus MCF7 was determined by imageJ and graphed. **(C)** MCF7 or MCF7/ADR cells were immunostained with anti CD44/CD24 antibody and were analyzed by FACS to determine the population of CD44+/CD24^-^ cells as a CSC marker. Total CD44^+^/CD24^-^ cells are represented as fold change against MCF7 in the bar graph. Values represent the mean ± SD from 3 independent experiments. **p < 0.01 compared with MCF7. **(D)** MCF7 and MCF7/ADR cells were treated with ALDEFLUOR or ALDEFLOUR DEAB reagent and total population of ALDH^+^ cells were analyzed by FACS. Bar graph represents total population of ALDH^+^ cells as fold change with respect to MCF7. Values represent the mean ± SD from 3 independent experiments. **p < 0.01 compared with MCF7. **(E)** MCF7 or MCF7/ADR cells were seeded on ultra-low attachment plates and the number of mammospheres developed was counted after 5 days. Bar graph represents number of mammospheres formed. Values represent the mean ± SD from 3 independent experiments. **p < 0.01 compared with MCF7. **(F)** qRT-PCR was performed to determine expression levels of CD44 in MCF7 and MCF7/ADR cells. Values represent the mean ± SD from 3 independent experiments. **p < 0.01 compared with MCF7. **(G)** CD44 and β-actin levels determined by immunoblot analysis of total cell lysate from MCF7 and MCF7/ADR cells.

To confirm the resistance of MCF7/ADR cells developed with continuous exposure of parental MCF7 cells to increasing concentration of doxo for several months, the MTT assay was performed. MCF7/ADR cells exposed to various concentration of doxo (1–16 μM) for 48 h and 72 h showed significantly high resistance compared to its parental cell line MCF7 (Supplementary Figure 1A and 1B). These doxo-resistant cell lines showed further cross-resistance to anticancer agent cisplatin (Supplementary Figure 1C) and reactive oxygen species (ROS) generating agent hydrogen peroxide (H_2_O_2_) (Supplementary Figure 1D). Resistance to doxo is predicted by enhanced expression of P-glycoprotein (P-gp) [40]. P-gp mRNA and protein level analysis revealed significant increase in MCF7/ADR cells compared to MCF7; P-gp mRNA level was more than 7000-fold higher (Figure 1A); similarly, the protein level was also observed to increase by ∼45-fold in MCF7/ADR cells compared to that in MCF7 (Figure 1B). Immunostaining for P-gp also revealed intense staining in MCF7/ADR cells compared to that in MCF7 cells (Supplementary Figure 1E). Drug-resistant cancer cells are reported to be associated with enrichment in CSCs [27]. To evaluate the CSC properties in MCF7 and doxo-resistant MCF7/ADR cells, we first evaluated the population of CD44^+^/CD24^-^ and ALDH^+^ cells, which have been reported as the best markers for identification of breast cancer stem cells [5, 15]. MCF7 and MCF7/ADR cells were stained with fluorescence-conjugated CD44 or CD24 and sorted by FACS. MCF7/ADR cells showed significantly higher number of cells with CD44^+^/CD24^-^ markers and ALDH^+^ activity, ∼40-fold higher CD44^+^/CD24^-^ populations compared to MCF7 (Figure 1C), and ∼5-fold higher ALDH activity compared to MCF7 (Figure 1D). As CSC-enriched populations have been reported to have a tendency to form spherical colonies in suspension cultures, termed tumor mammospheres [41], the mammosphere-developing ability of MCF7/ADR and MCF7 was evaluated by seeding single cell suspensions at concentrations (5000–15000 cells/well) in mammosphere culture media into low attached plates for 5 days. Mammospheres developed by MCF7/ADR cells were larger and ∼3-fold greater in number than those developed by MCF7 cells (Figure 1E). As reduced doxo resistance in CD44^+^/CD24^-^ breast cancer cells is evidenced by suppression of CD44 [42], we analyzed mRNA and protein levels for CD44; the result reveals MCF7/ADR cells with ∼150- and 16-fold higher levels of mRNA and protein, respectively, than in MCF7 (Figure 1F and 1G). These data suggest that doxo-resistant MCF7/ADR cells are enriched with CSCs with enhanced cancer stem cell properties.

### MCF7/ADR cells overexpress EXT1

**Figure 2:**
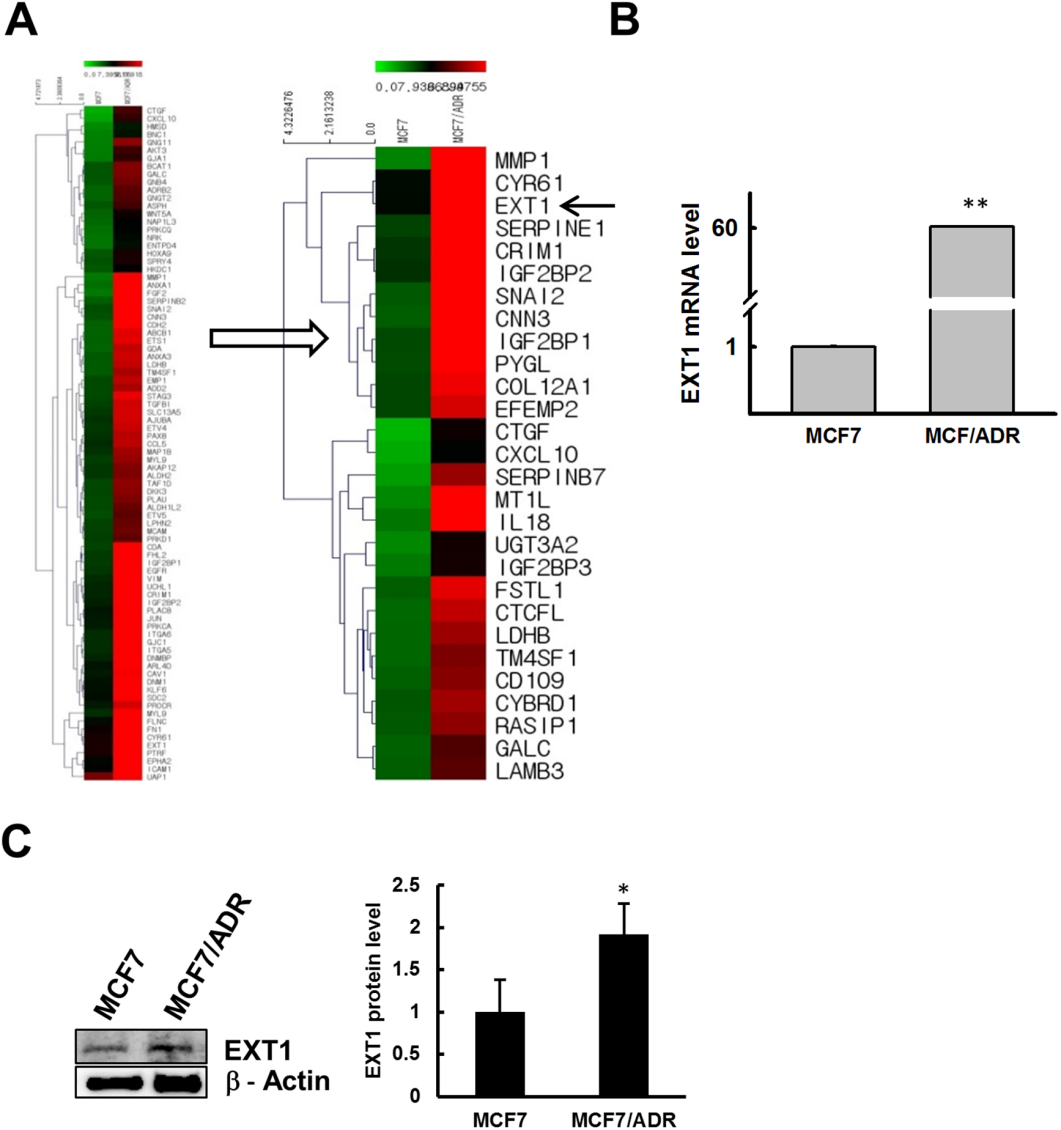
Doxo resistance enhances EXT1 expression in MCF7/ADR cells Microarray data analysis revealed large number of genes up or downregulated in MCF7/ADR cells, and EXT1 was selected as a candidate gene for further study. **(A)** Heat map showing several genes upregulated in MCF7/ADR cells. **(B)** EXT1 upregulation confirmed at the mRNA level by qRT-PCR and graphed after normalization with β-actin expression. **p < 0.01 compared with MCF7. **(C)** EXT1 protein level was determined by immunoblot analysis. *p < 0.05 compared with MCF7.

We next performed DNA microarray analysis using the Affymetrix Human Gene ST 1.0 Array system to analyze the differential gene expression profiles in MCF7 and MCF7/ADR cells. The expression of several hundreds of genes was up or down regulated in doxo-resistant MCF7/ADR than in MCF7 cells (Figure 2A). Among the most highly upregulated genes, we selected EXT1 for further analysis (Figure 2A), as EXT1 gene has been previously reported to have a potential role in cancer progression [38, 39]. We observed 24-fold higher EXT1 expression in MCF7/ADR by DNA microarray, which was verified by qRT-PCR and immunoblot analysis. qRT-PCR and immunoblot analysis for EXT1 in MCF7/ADR cells showed ∼60- and 1.9-fold higher expression for mRNA (Figure 2B) and protein (Figure 2C), respectively, compared to MCF7. Next, we asked whether an acute doxo treatment of MCF7 induces an alteration of EXT1 and P-gp. Tumors or cancer cells resistant to doxo are positive in P-gp staining [40]. Thus, we hypothesized that if the cells are unresponsive to doxo, then there should be increased levels of P-gp to eliminate doxo from the cells. MCF7 cells starved for 12 h and treated with vehicle or 3 μM of doxo for the next 12 h showed increased P-gp mRNA levels by ∼3.5-fold. At the same time, EXT1 mRNA was also found to be amplified by 3.4-fold (Supplementary Figure 2). These results indicate that short-term treatment of doxo in MCF7 cells can increase the P-gp level, and this adaptation may participate, in part, in the acquisition of doxo-mediated resistance.

### EXT1 promotes cancer cell stemness in MCF7/ADR cells

**Figure 3:**
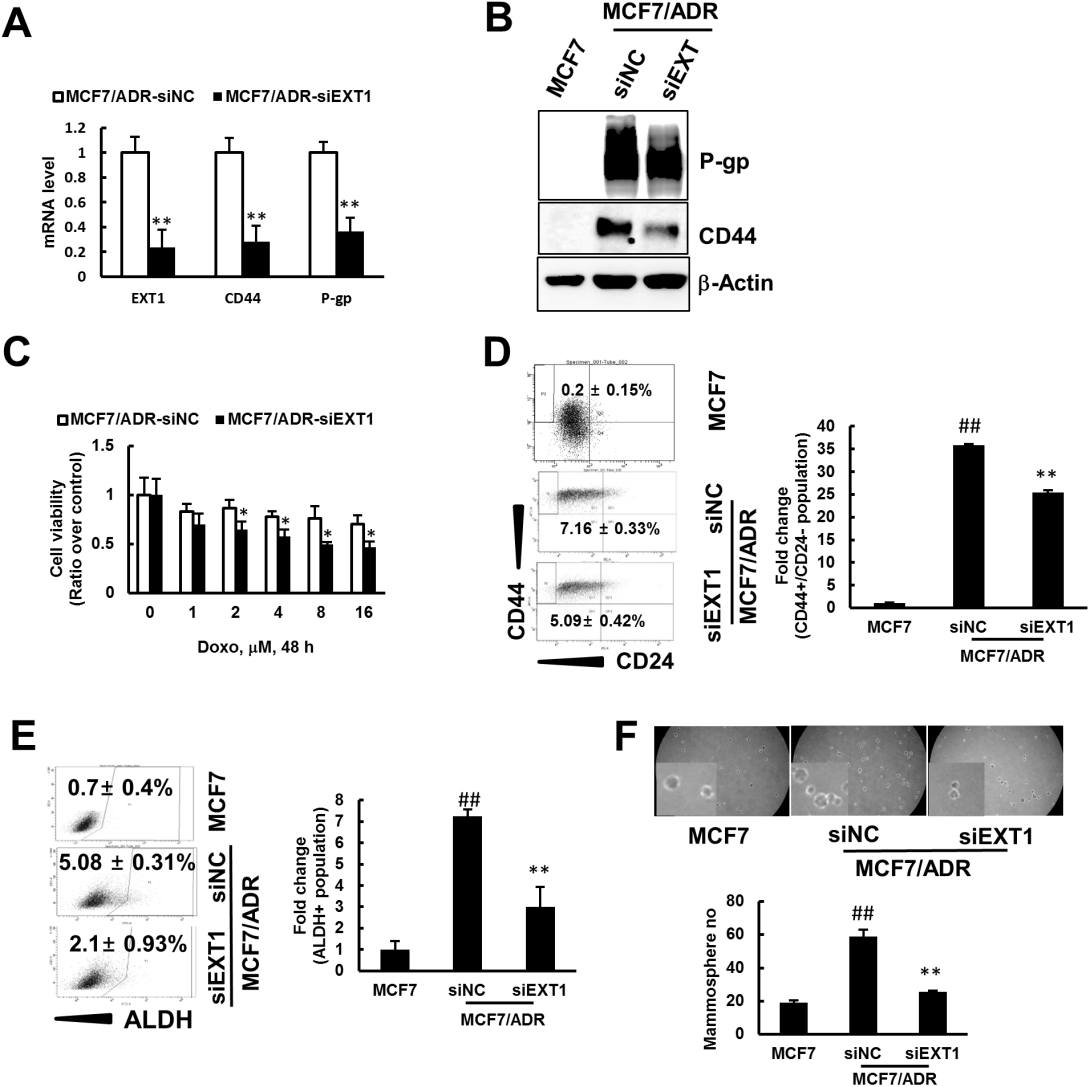
siRNA mediated knockdown of EXT1 represses cancer cell stemness and sensitization to doxo therapy Breast cancer cells, MCF7/ADR, were transfected with siEXT (MCF7/ADR-siEXT1) or negative control siRNA (MCF7/ADR-siNC) for 48 h. **(A)** Stem cell marker CD44 and resistant marker P-gp were determined by qRT-PCR. Data were graphed after normalization with β-actin expression level. Values represent the mean ± SD from 3 independent experiments. **p < 0.01 compared with MCF7/ADR-siNC. **(B)** CD44 and P-gp protein levels detected in MCF7, MCF7/ADR-siNC, and MCF7/ADR-siEXT1 by western blot analysis. **(C)** MCF7/ADR-siNC and MCF7/ADR-siEXT1 treated with doxo (0-16 μM) for 48 h and viable cells monitored by the MTT assay. Values represent the mean ± SD from eight wells. *p < 0.05 compared with MCF7/ADR-siNC. **(D)** MCF7, MCF7/ADR-siNC and MCF7/ADR-siEXT1 cells were analyzed for CD44+/CD24- population by FACS. Values represent the mean ± SD from 3 experiments. ^##^*p* < 0.01 compared with MCF7. **p < 0.01 compared with MCF7/ADR-siNC. **(E)** ALDH^+^ populations determined in MCF7, MCF7/ADR-siNC, and MCF7/ADR-siEXT1 by FACS analysis. Values represent the mean ± SD from 3 experiments. ^##^*p* < 0.01 compared with MCF7. **p < 0.01 compared with MCF7/ADR-siNC. **(F)** Single cell suspensions of MCF7, MCF7/ADR-siNC and MCF7/ADR-siEXT1 cells were grown on ultra-low attachment plates for 5 days and mammospheres developed were counted. Values represent the mean ± SD from 3 experiments. ^##^*p* < 0.01 compared with MCF7. **p < 0.01 compared with MCF7/ADR-siNC.

To study the possible role of EXT1 in promoting CSC properties in MCF7/ADR cells, siRNA-mediated knockdown of EXT1 was performed with two different constructs of siRNA targeting EXT1 (#1 and #2). EXT1 knockdown with both the constructs highly repressed mRNA and the protein level for EXT1 gene (Supplementary Figure 3A and 3B). Furthermore, as shown by immunocytochemistry (IC), MCF7/ADR cells with EXT1 knockdown (MCF7/ADR-siEXT1) revealed less staining with EXT1 antibody compared to MCF7/ADR cells transfected with negative control siRNA (MCF7/ADR-siNC) (Supplementary Figure 3C). Based on these results, we selected siEXT1 #1 for further investigation. We investigated the mRNA and protein expression level for CD44 and P-gp in the MCF7/ADR-siNC and MCF7/ADR-siEXT1 groups. MCF7/ADR-siEXT1 cells showed significantly decreased expression for CD44 and P-gp at both the mRNA and protein level compared to MCF7/ADR-siNC (Figure 3A and 3B). MCF7/ADR-siEXT1 showed approximately 60- and 70-fold repression at the mRNA level for P-gp and CD44, respectively, compared to MCF7/ADR-siNC (Figure 3A). Furthermore, the MTT assay revealed significantly increased cell death in response to doxo treatment in MCF7/ADR-siEXT1 compared to that in MCF7/ADR-siNC (Figure 3C). In addition, FACS analysis detected a significantly decreased CD44^+^/CD24^-^ population and repressed ALDH^+^ activity in MCF7/ADR-siEXT1 compared to that in MCF7/ADR-siNC (Figure 3D and 3E). Finally, in mammosphere culture, MCF7/ADR-siEXT1 cells developed fewer number of mammospheres compared to MCF7/ADR-siNC (Figure 3F). These results strongly support the hypothesis that increased EXT1 levels in drug-resistant MCF7/ADR cells is, in part, involved in the enrichment of CSC populations.

### EXT1 promotes EMT in doxo-resistant MCF7/ADR cells

**Figure 4:**
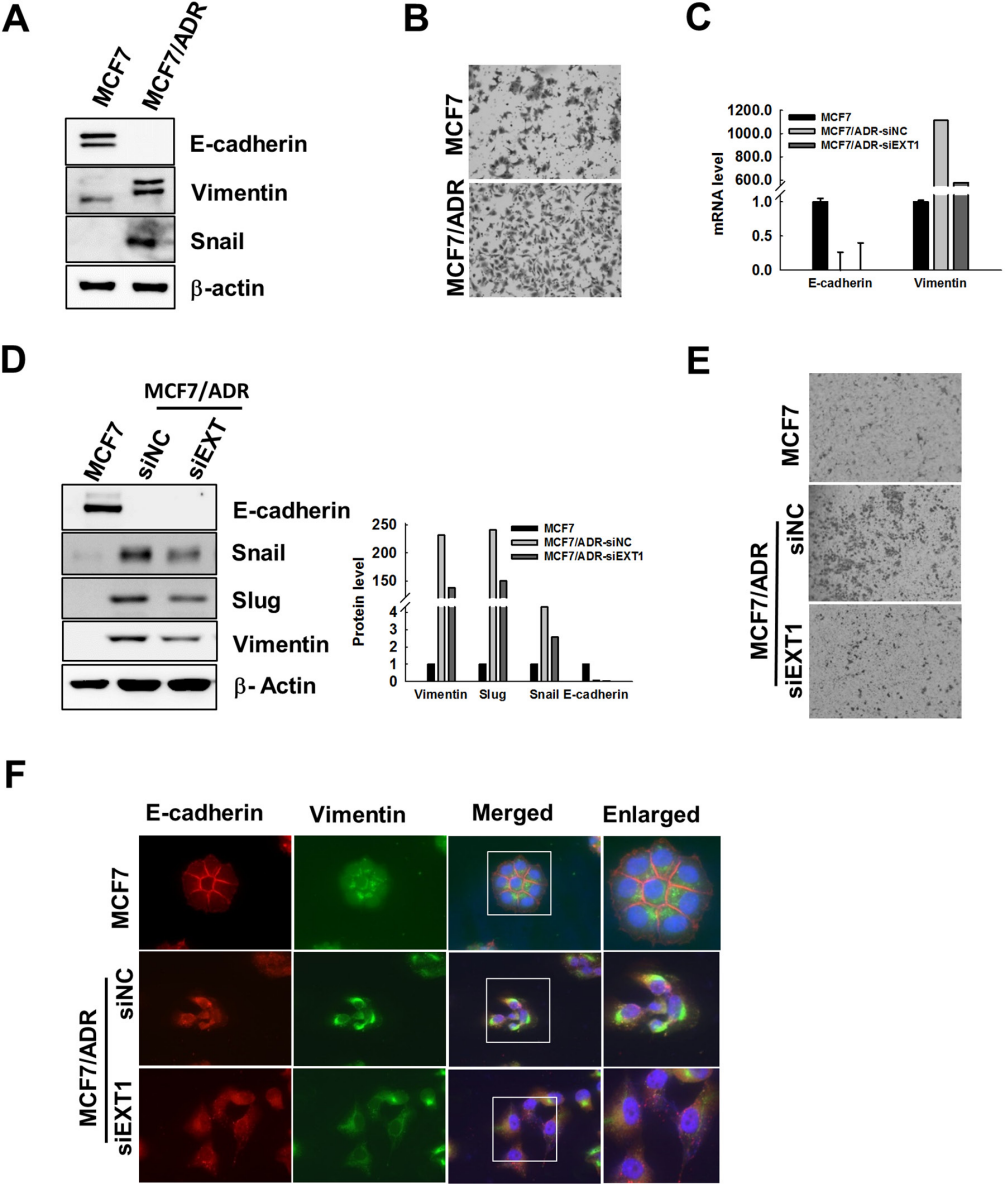
Knockdown of EXT1 represses EMT in MCF7/ADR cells **(A)** Protein lysates of MCF7 or MCF7/ADR cells were analyzed by immunoblot analysis for EMT marker proteins, vimentin, snail, and E-cadherin. **(B)** 4 × 10^4^ cells were seeded on the upper chamber of a transwell system in serum-free medium, and the lower chamber was loaded with 600 μL of complete medium, after 24 h cells migrated through the membrane were stained with hematoxylin and eosin. **(C-F)** Breast cancer cells, MCF7/ADR, were transfected with siEXT1 (MCF7/ADR-siEXT1) or negative control siRNA (MCF7/ADR-siNC) for 48 h. (C) mRNA level for E-cadherin and vimentin determined by qRT-PCR. (D) E-cadherin, vimentin, snail, and slug protein levels were determined in MCF7, MCF7/ADR-siNC and MCF7/ADR-siEXT1 cells by western blot analysis. Data were graphed after normalization with β-actin expression level. (E) Migratory efficacy of MCF7, MCF7/ADR-siNC, and MCF7/ADR-siEXT1 cells were determined by transwell migration assay. (F) Fluorescence immunostaining for E-cadherin and vimentin were imaged with a fluorescence microscope in MCF7, MCF7/ADR-siNC, and MCF7/ADR-siEXT1 cells.

Previous reports have revealed interrelation between EMT and an emerging chemo refractoriness of cancer cells to therapeutic intervention, followed by appearance of stem cell-like features [22, 27, 43]. In agreement with these reports, doxo-resistant MCF7/ADR cells were found to have overexpressed EMT markers, vimentin, and snail, compared to MCF7. In contrast, E-cadherin was reported to be repressed in CSC-enriched forms [44], and was under the limit of detection in MCF7/ADR cells (Figure 4A). In addition, transwell migratory efficacy was found to be highly facilitated in MCF7/ADR cells compared to that in MCF7 (Figure 4B). Thus, to clarify the role of EXT1 in the facilitation of EMT in MCF7/ADR cells, EXT1 was knocked down. MCF7/ADR-siEXT1 cells revealed repression of slug, snail, and vimentin, at both mRNA and protein levels, compared to MCF7/ADR-siNC (Figure 4C and 4D). In addition, E-cadherin was observed to be increased upon knockdown of EXT1 at the mRNA level (Figure 4C), but was undetected at the protein level (Figure 4D). Next, transwell migration assay migratory behavior of MCF7/ADR-siNC cells was abolished in MCF7/ADR-siEXT1 cells (Figure 4E). Furthermore, vimentin staining was precisely detected in MCF7/ADR-siNC compared to that in MCF7, which was found to be further reduced in MCF7/ADR-siEXT1 cells with EXT1 knockdown (Figure 4F). In contrast, E-cadherin was exclusively detected in MCF7 cells and was found to be significantly lower in MCF7/ADR-siNC cells, but MCF7/ADR-siEXT1 cells showed some positive staining for E-cadherin (Figure 4F). These data suggest that facilitated expression of EXT1 in doxo-resistant MCF7/ADR cells to enhance EMT is likely to be an underlying process of enriching cancer cell stemness.

### Overexpressed EXT1 in MCF7/ADR cell enhances cell surface heparan sulfate

**Figure 5:**
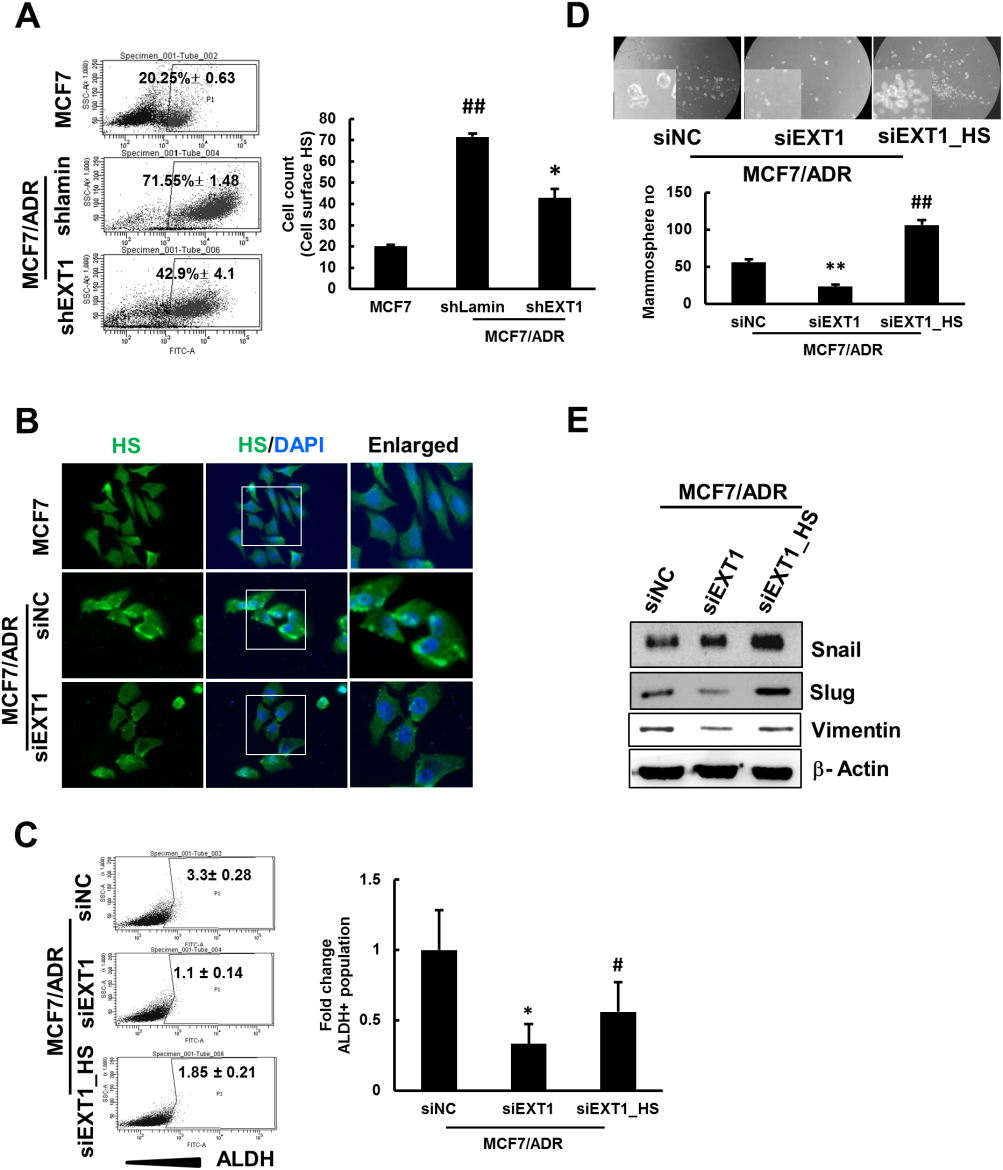
shRNA-mediated knockdown of EXT1 represses HS expression in MCF7/ADR cells, and HS supplement rescues MCF7/ADR cells from siEXT1 knockdown effects Breast cancer cells, MCF7/ADR, were transfected with shEXT1 (MCF7/ADR-shEXT1) or control shLamin (MCF7/ADR-shLamin) and selected for several weeks with blasticidin (5 μg/mL). **(A)** Cell surface HS were determined by FACS sorting in MCF7, MCF7/ADR-shLamin, and MCF7/ADR-shEXT1 cells. Values represent the mean ± SD from 3 experiments. ^##^*p* < 0.01 compared with MCF7. *p < 0.05 compared with MCF7/ADR-shLamin. Breast cancer cells, MCF7/ADR, were transfected with siEXT1 (MCF7/ADR-siEXT1) or control siNC (MCF7/ADR-siNC) for 48 h. **(B)** Immunostaining for cellular HS was visualized under a fluorescence microscope in MCF7, MCF7/ADR-siNC, and MCF7/ADR-siEXT1 cells. One group of MCF7/ADR cells maintained in HS-supplemented medium and transfected with MCF7/ADR cells maintained in normal medium with siEXT1 for 48 h, forming MCF7/ADR-siEXT1_HS and MCF7/ADR-siEXT1 or control siNC (MCF7/ADR-siNC). **(C)** ALDH^+^ populations determined in MCF7/ADR-siNC, MCF7/ADR-siEXT1, and MCF7/ADR-siEXT1_HS by FACS analysis. Values represent the mean ± SD from 3 experiments.^*^p < 0.05 compared with MCF7/ADR-siNC. #*p* < 0.05 compared with MCF7/ADR-siEXT1. **(D)** Single cell suspension of MCF7/ADR-siNC, MCF7/ADR-siEXT1, and MCF7/ADR-siEXT_HS cells were grown on ultra-low attachment plates for 5 days and mammospheres developed were counted. Values represent the mean ± SD from 3 experiments. ^**^p < 0.01 compared with MCF7. ##*p* < 0.01 compared with MCF7/ADR-siEXT1. **(E)** Snail, slug, and vimentin protein levels were determined in MCF7/ADR-siNC, MCF7/ADR-siEXT1, and MCF7/ADR-siEXT1_HS cells by western blot analysis.

EXT1 is involved in the biosynthesis of HS [34] and HS is reported to be involved in various developmental and inflammatory processes [45, 46]; however, its role in carcinogenesis and cancer cell stemness is not known. First, we confirmed if HS can facilitate doxo resistance in MCF7 cells. MCF7 cells continuously maintained in media supplemented with HS were treated with various concentrations of doxo for 48 h. In each of the treatment groups, the cells showed increased viability than that of MCF7 cells grown in normal media lacking HS, revealing a possible involvement of HS in enhancing chemoresistance (Supplementary Figure 4). To verify the involvement of EXT1 in doxo-resistant human breast cancer stemness through biosynthesis of HS, EXT1 knockdown cells were analyzed by FACS to detect cell surface HS. MCF7/ADR-shLamin showed more than 3-fold numbers of cells with cell surface HS compared to MCF7. MCF7/ADR-shEXT1 cells were reduced to ∼40% of cells with cell surface HS compared to MCF7/ADR-shLamin (Figure 5A). Similarly, immunostaining with anti-HS antibody also revealed that MCF7/ADR-siEXT1 cells acquired lesser fluorescence intensity than MCF7/ADR-siNC (Figure 5B). In addition, to confirm that EXT1-mediated enrichment of cancer cell stemness in MCF7/ADR is specifically due to increased cell surface HS, MCF7/ADR cells were maintained in HS-containing media for 15 days before the experiments. MCF7/ADR cells maintained in HS or HS-free media were transfected with siEXT1, to transiently knockdown EXT1, or siNC, as control. MCF7/ADR-siEXT1 cells maintained in normal media showed a 3-fold decrease in the ALDH^+^ population compared to MCF7/ADR-siNC. As a recovery function, MCF7/ADR-siEXT1 cells maintained in HS supplemented media showed a ∼1.6-fold increase in the ALDH^+^ population when compared to MCF7/ADR-siEXT1 cells grown in HS-free media (Figure 5C). Similarly, mammospheres grown in MammoCult™ and protein levels of EMT-associated genes were also increased in HS-supplemented cells as compared to that in EXT1-knockdown cells maintained in normal media (Figure 5D and 5E). These results indicate a recovery function of HS upon EXT1 knockdown and strongly support the finding that enrichment of cancer cell stemness in MCF7/ADR cells might be mediated through EXT1 by boosting the HS biosynthesis machinery.

### Stable knockdown of EXT1 reduces cell proliferation and colonies on soft agar *in vitro*

**Figure 6:**
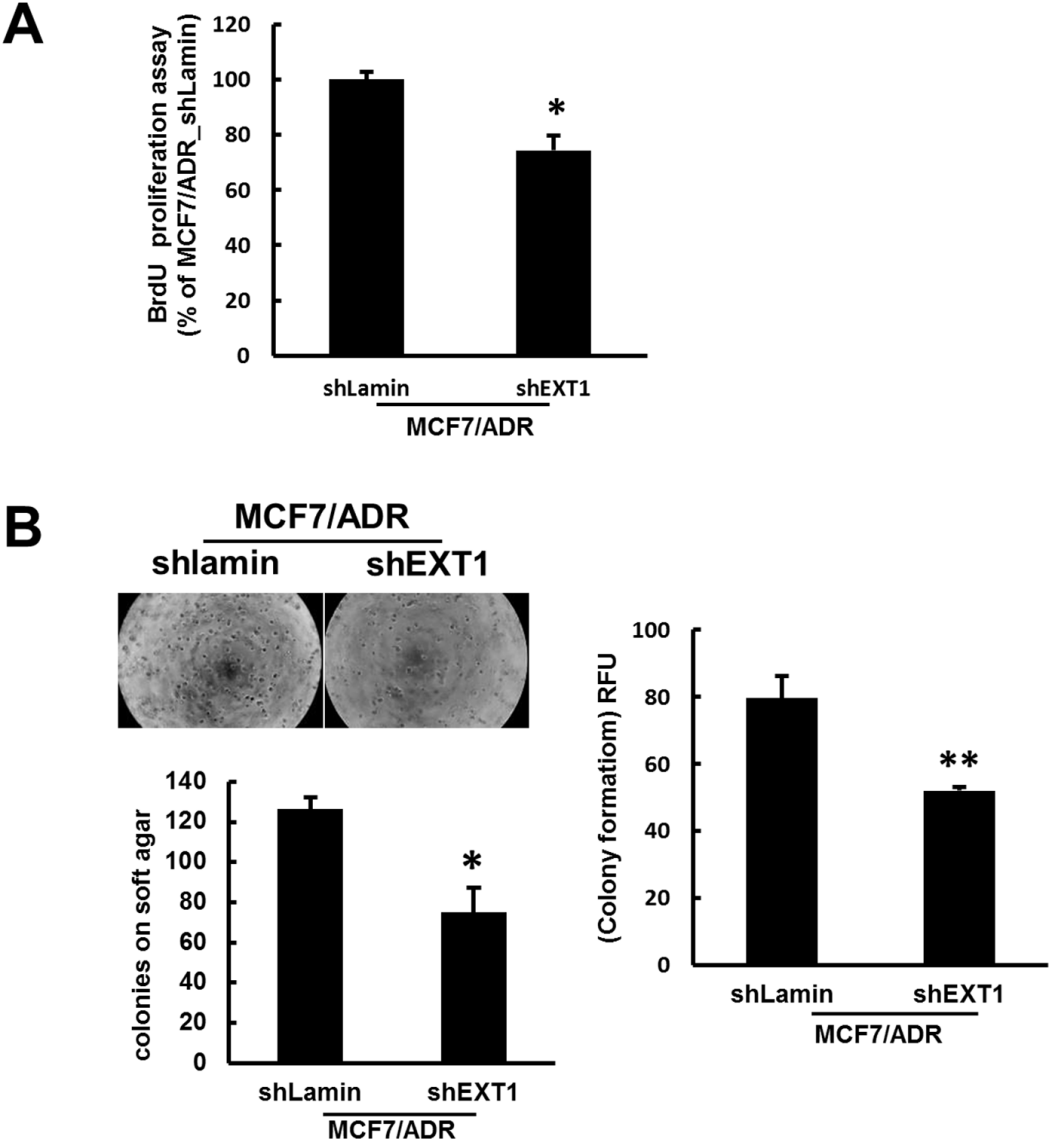
shRNA-mediated inhibition of EXT1 represses cell proliferation and *in vitro* anchorage-independent growth on soft agar **(A)** Proliferation of MCF7/ADR-shLamin and MCF7/ADR-shEXT1 cells determined with the BrdU proliferation assay kit. Values represent the mean ± SD from 3 experiments. ^*^p < 0.05 compared with MCF7/ADR-shEXT1. **(B)** Anchorage-independent growth of MCF7/ADR-shLamin and MCF7/ADR-shEXT1 cells determined with the CytoSelect 96-well Cell Transformation Assay kit. Values represent the mean ± SD from 3 experiments. ^**^p < 0.05 compared with MCF7/ADR-shEXT1.

To confirm the potency of EXT1 as a tumor promoter in MCF7/ADR cells, MCF7/ADR-shEXT1 or MCF7/ADR-shLamin control cells were established with transfection of shEXT1 or shLamin plasmid, respectively, followed by selection with blasticidin. Repressed EXT1, together with reduced expression for EMT marker genes in MCF7/ADR-shEXT1 cells, was confirmed (Supplementary Figure 5). Stable knockdown of EXT1 reduced cell proliferation by 25% (Figure 6A). *In vitro* anchorage-independent growth, which is defined as the ability of transformed cells to grow independently on a solid surface, is an indication of metastatic potency of tumors [47]. We performed a colony formation assay on soft agar, which revealed a 40% decrease in colony number and relative fluorescence units (RFUs) in the EXT1 knockdown group as compared with that in the control group (Figure 6B). These results verify the potentiality of EXT1 as a tumorigenic unit.

### Overexpressed EXT1 in MCF10A enriches cancer stem cell-like properties

**Figure 7:**
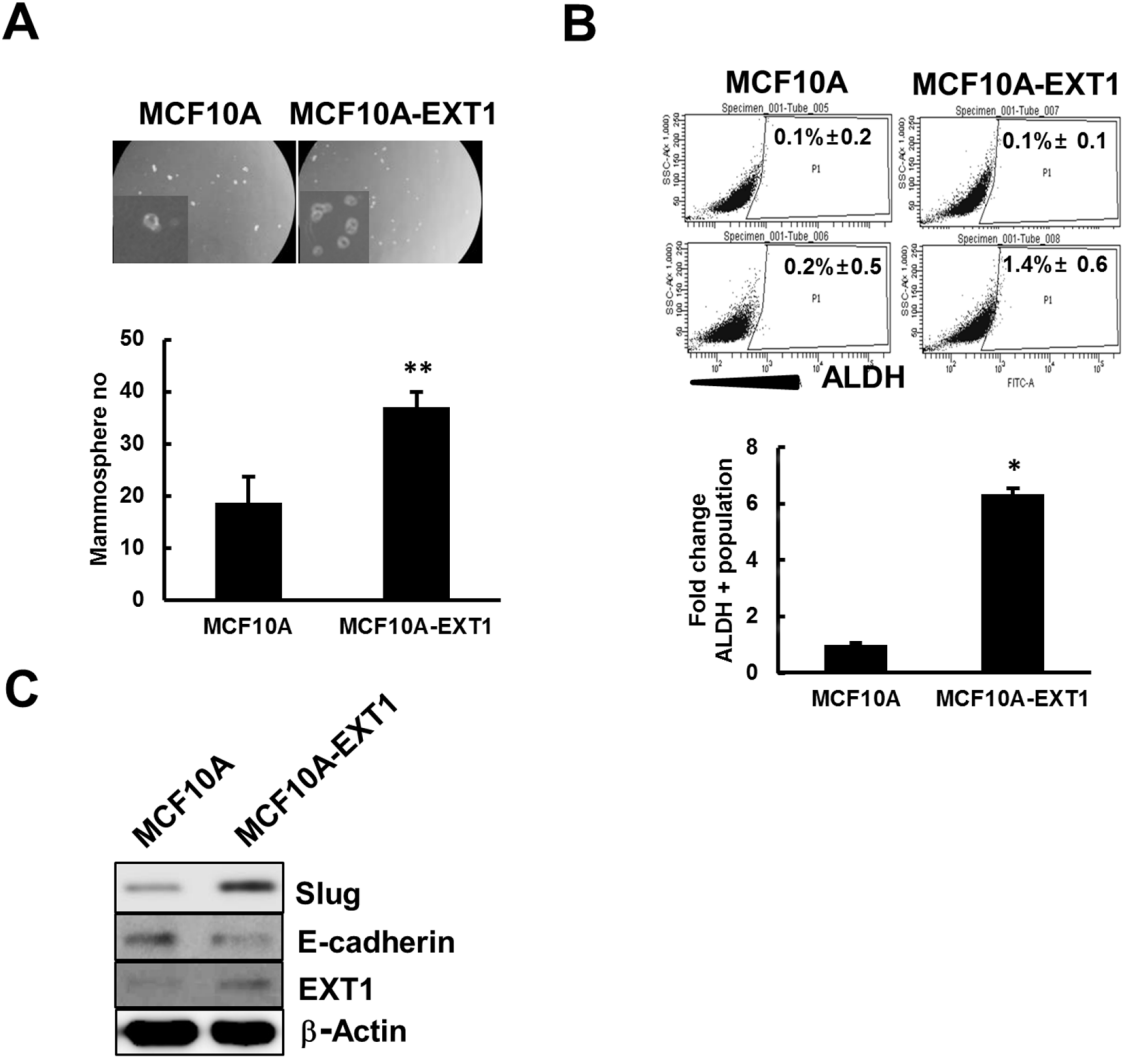
EXT1 overexpression in normal breast cells, MCF10A, increased stem cell like properties MCF10A cells transfected with blank vector (MCF10A) or vector with human *EXT1* gene insertion. (MCF10A-EXT1) for 48 h. **(A)** Single cell suspension of MCF10A and MCF10A-EXT1 cells were grown on ultra-low attachment plates and mammospheres developed were counted after 5 days. Values represent the mean ± SD from 3 experiments. **p < 0.01 compared with MCF10A. **(B)** ALDEFLUOR or ALDEFLOUR DEAB treated MCF10A and MCF10A-EXT1 cells were analyzed for total population of ALDH+ cells. Bar graph represents ALDH+ cells as fold change with respect to MCF10A. Values represent the mean ± SD from 3 independent experiments. *p < 0.05 compared with MCF10A **(C)** Slug, E-cadherin, and EXT1 proteins were determined in MCF10A and MCF10A-EXT1 cells by western blot analysis.

To confirm that overexpression of EXT1 can transform normal breast epithelial cells to the malignant form, we overexpressed EXT1 in MCF10A cells (Supplementary Figure 6) and these cells (MCF10A-EXT1) were evaluated for their mammosphere-generating and ALDH^+^ population-enriching capability. MCF10A-EXT1 cells generated 2-fold more mammospheres compared to MCF10A (Figure 7A). Similarly, in FACS analysis, MCF10A-EXT1 cells revealed 6-fold high numbers of ALDH^+^ cells than MCF10A cells transfected with blank vector (Figure 7B). Furthermore, levels of EMT marker proteins slug and E-cadherin were increased and decreased, respectively, with overexpression of EXT1 in MCF10A cells (Figure 7C). These results suggest that overexpression of the single molecule EXT1 can transform normal cells to the malignant form by facilitating cancer stemness-like properties.

## DISCUSSION

Enrichment of CSCs within tumors in response to chemotherapy is the most critical issue hindering the overall survival of patients with breast cancer. This suggests that many cancer therapies fail because chemotherapy cannot eliminate CSCs, which ultimately grow to form new more aggressive tumors [16, 18]. Hence, to overcome the burden of tumor relapse and therapeutic resistance, CSCs are currently of great interest to cancer researchers. In this study, we report that CSC-like features are increased in response to hyperactivation of EXT1 in MCF7/ADR, a doxo-resistant breast cancer cell line, than in MCF7, a parental cell line. These transformed cells not only showed CSC enrichment but also had overexpressed P-gp, facilitated EMT, and higher resistance to chemotherapy.

However, EXT1 is well-defined as a tumor suppressor in benign bone tumors [36], ER-negative tumors, and tumors of patients with successively developed distant metastasis have high levels of EXT1 expression [48]. In addition, tumorigenic behavior of EXT1 is indicated in HL-60 cells by a marked reduction in colony formation and xenograft implantation upon repression of EXT1 [49]. These reports imply a tissue- or cell-type specific role of EXT1 as a suppressor or promoter of cancer growth. We validated EXT1 as a tumor promoter with an siRNA-mediated approach in MCF7/ADR cells, and revealed its novel role as a regulator of CSC properties. EXT1 regulates HS biosynthesis and the role of HS is extended from formation of blood vessels to the involvement in the progression of metastasis [50]. It is well-known that HS can regulate self-renewal and pluripotency in embryonic stem (ES) cells [51] and similar signaling pathways are associated in regulating the self-renewal process of both stem cells and CSCs [18]. Thus, our finding could be of specific importance to the management of CSC properties in breast cancer cells exposed to doxo.

The cytotoxic effect of chemotherapy is overcome through several mechanisms, including enhanced ABC transporters, facilitated ALDH activity, and overexpressed B-cell lymphoma-2 (BCL2) [52].Exposure to doxo leads to increased multi drug resistance (MDR) phenotype in cancer cells, with cross-resistance to chemotherapeutic agents correlating with overexpressed P-gp [53, 54]. However, knockdown of P-gp has no direct effect on stem cells, revealing its insignificance in stemness growth, but it can still protect cells from the cytotoxic effect of chemotherapy [55]. In clinical trials, the use of verapamil, an inhibitor of P-gp, to increase efficacy of chemotherapy was limited due to its toxic side effect [56]. Thus, it is likely more effective to directly target the molecule augmenting P-gp to overcome enrichment of CSCs within tumors. In this study, we illustrated EXT1 as a candidate to increase P-gp, indicating its possible role in the enrichment of CSCs. Acute treatment of doxo in MCF7 increased mRNA levels for both EXT1 and P-gp (Supplementary Figure 2), and knockdown of EXT1 in doxo-resistant MCF7/ADR cells repressed expression for P-gp, thus sensitizing these cells to doxo-mediated toxicity (Figure 3A, 3B and 3C). Hence, we advocate that targeting EXT1 could be a promising novel approach for overcoming chemotherapeutic side effects and managing enrichment of CSCs in clinical trials with patients with therapeutic resistance to doxo.

Hepatocellular carcinoma (HCC) cells exhibited EXT1-mediated activation of TGF-β-enhancing chemosensitivity to 5-FU [57], implicating its role in the augmentation of EMT features. TGF-β is associated with CSC enrichment, metastasis, and survival against chemotherapy [22, 25, 27]. Here, we underscored hyperactivated EXT1 in MCF7/ADR cells for increased EMT, which was further repressed with knockdown of EXT1, additionally decreasing CSC properties. Overall, our results illustrate the novel role of EXT1 as an EMT promoter, eventually resulting in a CSC-enriched phenotype in the doxo refractory breast cancer cell line, MCF7/ADR.

**Figure 8:**
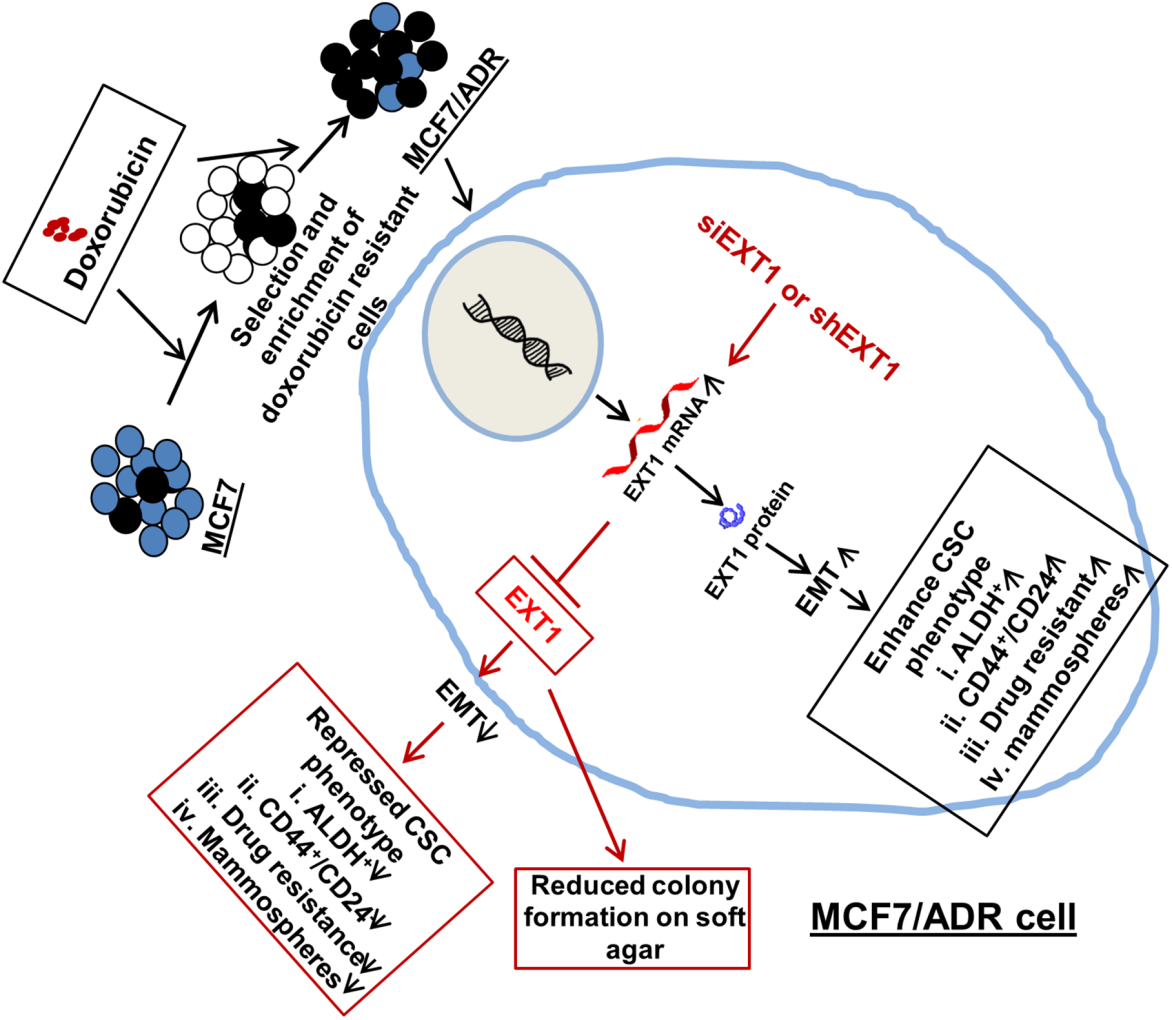
Proposed mechanistic model for cancer cell stemness regulated by EXT1 in MCF7/ADR cells Enhanced EXT1 in doxo-resistant MCF7/ADR cells facilitated EMT and enhanced cancer cell stemness. With the knockdown of EXT1, EMT was repressed, thus suppressing cancer cell stemness and sensitization to doxo therapy. Furthermore, anchorage-independent growth on soft agar was abrogated with knockdown of overexpressed EXT1 in MCF7/ADR cells.

Collectively, we demonstrated the critical role of enhanced EXT1 in regulating cancer cell stemness in doxo-resistant MCF7/ADR cells. In particular, we identified that MCF7/ADR cells with highly expressed EXT1 increased EMT and cancer cell stemness, thus facilitating anchorage-independent colony formation in soft agar. Consequently, siRNA-mediated knockdown of EXT1 in MCF7/ADR cells abolished cell surface HS. Furthermore, repressed EMT signatures, reduced cancer stem cell features, and sensitization of these cells to doxo-mediated toxicity were observed (Figure 8). In addition, doxo is utilized to treat breast cancer at different stages, including advanced stage, before surgery to shrink tumors, or after surgery to reduce the risk of recurrence. As our research has illustrated that doxo treatment can enrich CSCs with increased EXT1 protein within breast cancer cells, it is possible that tumors grow more aggressively with increased translation of EXT1 protein upon exposure to doxo. Hence, these findings may provide a new insight into EXT1 as a promising novel therapeutic target for overcoming breast cancer stemness and chemoresistance in breast cancer patients with anthracycline-based therapeutic resistance.

## MATERIALS AND METHODS

### Reagents

Antibodies recognizing CD44, snail, slug, vimentin, and P-gp were purchased from Cell Signaling technologies (Danvers, MA, USA), E-cadherin was purchased from Abcam (Cambridge, MA, USA), EXT1, β-actin was purchased from Santa Cruz Biotechnology (Santa Cruz, CA, USA), and HS was purchased from Amsbio (Abingdon, UK). Doxo and DAPI were purchased from Sigma-Aldrich (Saint Louis, MO, USA). Methylthiazoletetrazolium solution (MTT) was obtained from AMRESCO (Solon, OH, USA). M-MLV Reverse Transcriptase was purchased from Promega (Madison, WI, USA) and VeriQuest SYBR Green qPCR Master Mix was obtained from Affymetrix (Cleveland, OH, USA). Pro-PREP protein extraction solution was purchased from iNtRON Biotechnology (Seongnam, Korea). Fluorochrome-conjugated monoclonal antibodies against human CD44 (FITC; cat. #555478) and CD24 (PE; cat. #555428) or the respective isotype controls were purchased from BD Biosciences (San Diego, CA, USA). ALDEFLUOR™ kit was obtained from STEMCELL Technologies (Vancouver, Canada). Predesigned siRNA targeting EXT1 (siEXT1) and negative siRNA (siNC) were from BIONEER Co (Daejeon, South Korea).

### Cell culture and establishment of MCF7/ADR cells

MCF7, human breast cancer cells, were obtained from ATCC and maintained in DMEM (Hyclon, Logan, UT, USA) containing 10% FBS (Hyclon) and 1% penicillin/streptomycin (10,000 U/mL). MCF7/ADR cells were developed by continuous incubation and selection of MCF7, breast cancer cells in the laboratory of Dr. Keon Wook Kang, Seoul National University, maintained in complete media with increasing concentration of doxo up to 1 μM. MCF7/ADR cells were used for further experimental process after they showed stability of resistance after incubation in doxo-free normal medium for a week. Normal human breast epithelial cells, MCF10A, were maintained in DMEM/F12 media supplemented with insulin (10 μg/mL), cholera toxin (100 ng/mL) (Sigma-Aldrich), EGF (20 ng/mL) (R&D systems, Wiesbaden, Germany), hydrocortisone (500 ng/mL), and L-glutamine (Invitrogen, Gaithersburg, MD, USA).

### MTT assay

Fractions of viable cells were determined through MTT assay. MCF7 cells were seeded at a density of 5 × 10^3^ cells/well in 96-well plates and incubated with the relevant compounds (doxo, H_2_O_2_, or cisplatin) for the indicated times. After completion of incubation time, 0.5 mg/mL MTT was added to the cells and further incubated at 37°C for 4 h. The MTT solution was removed, DMSO (100 μL/well) was added, and the absorbance was measured at 540 nm using an Infinite M200 Pro (Tecan Group Ltd., Männedorf, Switzerland).

### DNA microarray analysis

Total RNAs were isolated from MCF7 and MCF7-ADR cells using the Totally RNA kit (Ambion, Austin, TX, USA). Isolated total RNAs were purified using the RNeasy Mini kit (Qiagen, Valencia, CA, USA) and used for cDNA synthesis (Invitrogen) and cRNA labeling (Enzo Biochemical, Farmingdale, NY, USA). Fragmented cRNAs (12.5 μg) were hybridized at 45°C for 16 h to a Human Gene ST array (HuGene-1-0-ST-v1; Affymetrix, Santa Clara, CA, USA). Finally, data were generated with GenPlex version 3.0 software (ISTECH, Korea).

### Total RNA extraction and quantitative real-time PCR (qRT-PCR)

Total RNA was extracted using TRIzol reagent (Invitrogen) and first strand cDNA was synthesized by means of MMLV reverse transcriptase according to the manufacturer’s protocol. Quantitative real-time PCR was performed using the Bio-Rad CFX96TM Real-Time System PCR Cycler (Bio-Rad Laboratories, Hercules, CA, USA) with VeriQuest SYBR Green qPCR Master Mix. The PCR program consisted of an initial denaturation step at 95°C for 10 s, followed by 40 cycles of 95°C for 5 s and 60°C for 1 min. The expression level of each specific gene was normalized against the expression of actin in the same master reaction. The primers for P-gp were 5′-TGGGAAGATCGCTACTGAAGC-3′ and 5′-TTTCCTCAAAGAGTTTCTGTATGGTA-3′; vimentin, 5′-TGTCCAAATCGATGTGGATGT-3′ and 5′-TTGTACCATTCTTCTGCCTCC-3′; E-cadherin, 5′-GAGAGCGGTGGTCAAAGAGC-3′ and 5′-GAGTTCAGGGAGCTCAG-3′; and for Actin were 5′-GGATGTCCACGTCACACTTC-3′ and 5′-CACTCTCCAGCCTTCCTTC-3′. Bioneer predesigned primers were used for EXT1 and CD44 with catalog numbers P237660 and P170666, respectively (Bioneer, Daejeon, Korea). All the experiments were performed in triplicate and the average was calculated.

### Immunoblot analysis

Cells were washed twice with cold PBS and followed by lysis with the addition of ice-cold Pro-PREP protein extraction solution. The lysates were briefly vortexed and incubated on ice and insoluble components were removed by centrifuging at 15,000 × *g* at 4°C for 20 min. Protein concentrations were determined using the BCA Protein Assay Kit (Thermo Scientific, Rockford, IL, USA). Aliquots of 25–30 μg of proteins were resolved on 8–12% SDS-PAGE gels and transferred to nitrocellulose membranes (Whatman, Dassel, Germany). Membranes were blocked with 5% skimmed milk and immunoblotted with antibodies at a dilution of 1:500. Following addition of enhanced chemiluminescence (PIERCE, Woburn, MA), images were visualized using an ImageQuant™ LAS 4000 imager (GE Healthcare Ltd., Buckinghamshire, UK).

### Stem cell detection

For the detection of populations of stem cells enriched with cell surface marker CD44^+^/CD24^-^, cells were harvested by trypsinization. 10^6^cells/mL was re-suspended in PBS supplemented with 0.1% bovine serum albumin (BSA). Cells were then incubated with fluorochrome-conjugated monoclonal antibodies against human CD44 (FITC) and CD24 (PE) or the respective isotype controls for 1 h at 4°C. The labeled cells were sorted and analyzed on a BD FACS AriaIII cell sorter (BD Biosciences).

### ALDEFLUOR assay and flow cytometry

To detect ALDH activity, the ALDEFLOUR assay was carried out according to the manufacturer’s protocol. Briefly, 10^6^ cells were incubated in Aldefluor assay buffer containing an ALDH substrate, bodipy-aminoacetaldehyde (BAAA), at a concentration of 1.5 μM for 45 min at 37°C. A fraction of cells were incubated with a 10-fold molar excess of an ALDH inhibitor, diethylaminobenzaldehyde (DEAB), to use as a negative control. The cells were sorted and analyzed on a BD FACS AriaIII cell sorter (BD Biosciences) for ALDH activity.

### Mammosphere assay

Single-cell suspensions were plated in 6-well ultralow attachment suspension culture plates (Corning CoStar) at a density of 1,000 to 15,000 viable cells/well. Cells were grown in 2 mL of MammoCult™ Human Medium kit (STEMCELL Technologies) and mammospheres were counted after 5 days.

### siRNA transfection

Pre-designed and pre-annealed siRNAs targeting EXT1 or the negative control, and scrambled siRNAs were purchased from Bioneer Corporation (Daejeon, Korea). Briefly, 3 × 10^3^ or 4 × 10^5^cells were plated in a 96-well plate or 60-mm disc, respectively. On the next day, cells were transfected with 50 nM of siRNA targeting EXT1 (siEXT1) or a negative control siRNA (siNC) using HiPerFect transfection reagent (QIAGEN GmbH, Hilden, Germany) according to the manufacturer's instructions and further incubated for 48–72 h depending on experimental design. The sequences for siEXT1#1 and #2 or siNC are as listed in Table 1 below.

**Table 1:** siRNA sequences

siRNA	Sequences
siEXT1#1	Sense: 5′-CACUUCUGGGAUAACUCUA-3′
	Antisense: 5′-UAGAGUUAUCCCAGAAGUG-3′
siEXT1#2	Sense: 5′-UGUUCGUACUACCACAGUA-3′
	Antisense: 5′-UACUGUGGUAGUACGAACA-3′
siNC	Sense: 5′-CUGAUGACCUGAGUGAAUG-3′
	Antisense: 5′-CAUUCACUCAGGUCAUCAG-3′

### EXT1 cloning, cell culture, and transfection

The human *EXT1* gene was amplified via PCR from a human placental cDNA library. The resulting fragment was ligated into the pCS4 vector and the insert integrity was verified by sequencing. The human normal breast epithelial cell line MCF10A was grown in DMEM/F12 media supplemented with 5% fetal bovine serum (FBS), 10 μg/mL insulin, 100 ng/mL cholera toxin (Sigma-Aldrich), 20 ng/mL rEGF ((R&D systems, Wiesbaden, Germany), 500 ng/mL hydrocortisone, and L-glutamine (Invitrogen) at 37 °C under 5% CO_2_. The plasmids were transfected using the Lipofectamine™ 2000 reagent according to the manufacturer's instructions.

### Transwell migration assay

The migratory activity of breast cancer cells was analyzed through the transwell system (6.5 mm diameter, 8 μM pore size, Corning Costar). Briefly, cells were plated on the upper chamber of transwells at the density of 1 × 10^4^ cells in 100 µL of serum-free DMEM, and complete medium was added to the lower chamber. After 24 h of incubation at 37°C, cells were fixed with methanol followed by staining with hematoxylin and eosin, and counted under a stereo microscope (Carl Zeiss™ Stemi 2000-C). Three independent experiments were performed.

### Immunofluorescence staining

Cells were seeded on to adhesion cover slips for 24 h. The next day they were fixed with 4% paraformaldehyde in PBS for 10 min at 25°C and permeabilized with 0.1% Triton X-100 in PBS. Cells were then blocked with 1% BSA for 30 min and incubated overnight at 4°C with antibodies to EXT1, vimentin, E-cadherin, or heparan sulfate at a dilution of 1:200, followed by 1 hr incubation with secondary antibodies AlexaFluor 568- or Alexa 488-conjugated IgG (Life Technologies, Carlsbad, CA, USA) at 1:200. DAPI was used for nuclear staining and cells were mounted was in Fluoromount™ aqueous mounting medium (Sigma-Aldrich). Samples were visualized using a fluorescent Axio Imager A1 and AxioVision (Carl Zeiss Imaging Systems, Jena, Germany) software (200X magnification).

### Generation of stable EXT1 knockdown cell line

For the stable knockdown experiments, EXT1 shRNA stable transfectants were established by synthesized shEXT1 oligonucleotides harboring the EXT1 shRNA sequence and cloning them into a blasticidin-resistant pBLOCK-iT™ Gateway vector (Invitrogen), according to the manufacturer’s protocol. pBLOCK-iT™-GW/U6-laminshRNA Vector (Invitrogen) was used as a control vector. shEXT1 or shlamin were transfected into MCF7/ADR cells with Lipofectamine™ 2000 according to the manufacturer’s instructions (Invitrogen). shRNA transfectants were selected by treating cells with 5 µg/mL blasticidin for 21 days. The sequence for the shEXT1 is 5'-GCGACAGAGCTGCATGAATAC-3'.

### Soft agar colony formation assay

Anchorage-independent growth of cells was determined using the CytoSelect 96-well Cell Transformation Assay kit (Cell Biolabs, San Diego, CA, USA) by performing soft agar colony formation assays. The numbers and morphologies of colonies were determined using an inverted phase-contrast microscope (Olympus, Tokyo, Japan) after 10 days of seeding. To quantify the anchorage-independent growth, colonies were lysed with lysis buffer and CyQuant GR dye was added to generate fluorescence. Finally, fluorescence was measured using a fluorometer with filter setting at 485/520 (Wallac Victor3 1420 mutilabel counter, Perkin Elmer, Waltham, MA, USA). Data are presented as the means ± SD of 3 independent wells.

### Statistical analysis

The Student’s t-test was performed to assess the significances of differences between experimental groups. Differences with *p* < 0.05 and *p* < 0.01 were considered statistically significant and highly significant, respectively. All experiments were performed in triplicate and the data are expressed as the means ± SD.

## SUPPLEMENTARY MATERIALS FIGURES


