# Supplementary material for: Exostosin 1 regulates cancer cell stemness in doxorubicin-resistant breast cancer cells
Source: Oncotarget. 2017 Jul 31;8(41):70521–37. doi: 10.18632/oncotarget.19737 (PMC5642574; doi:10.18632/oncotarget.19737)
Supplement: Supplementary file 1 [file oncotarget-08-70521-s001.pdf]

Exostosin 1 regulates cancer cell stemness in doxorubicin-resistant breast cancer cells

SUPPLEMENTARY MATERIALS

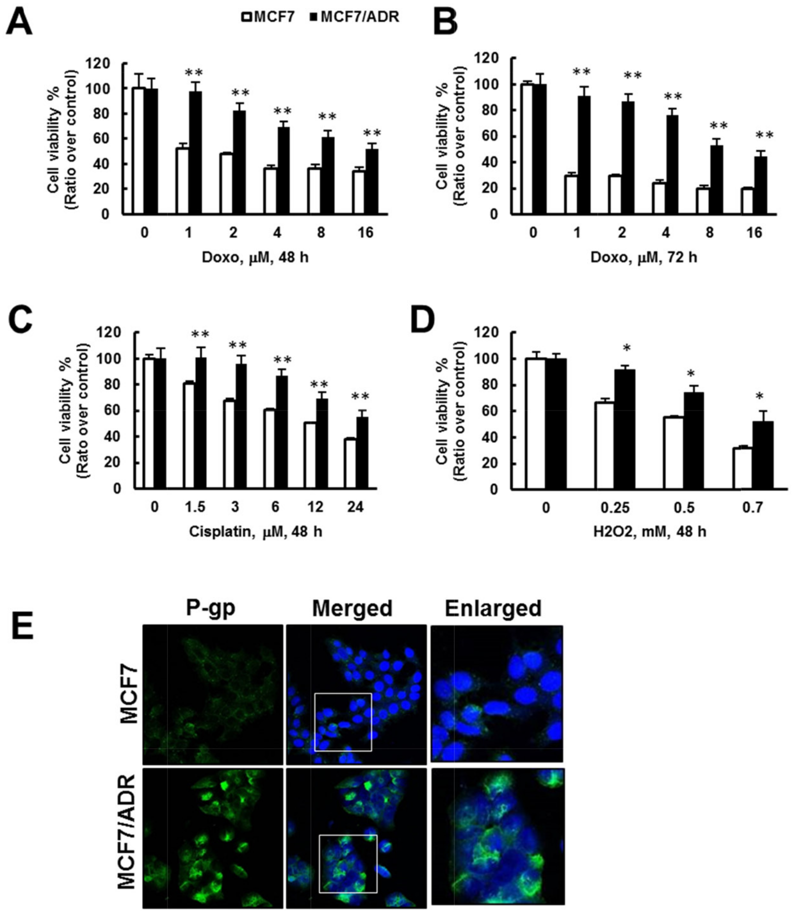

Supplemenatry Figure 1: Doxo treatment for extended time enhances resistance of MCF7 cells. **(A)** and **(B)** MCF7 and MCF7/ADR cells were treated with various concentrations of doxo (2–16 µM) for 48 or 72 h and the MTT assay was performed. Values are means ± SD from 8 samples. Data are expressed as ratio to vehicle control. $^{**}p < 0.01$ compared with MCF7. **(C)** MCF7 and MCF7/ADR cells treated with various concentration of cisplatin (2-24 µM). $^{**}p < 0.01$ compared with MCF7 or **(D)** H_2O_2 (0.3 to 1 mM) for 48 h and MTT assay was performed. Values are means ± SD from 8 samples. Ratio over vehicle control. $^{*}p < 0.05$ compared with MCF7. **(E)** P-gp immunostaining was detected in MCF7 and MCF7/ADR cells with a fluorescence microscope.

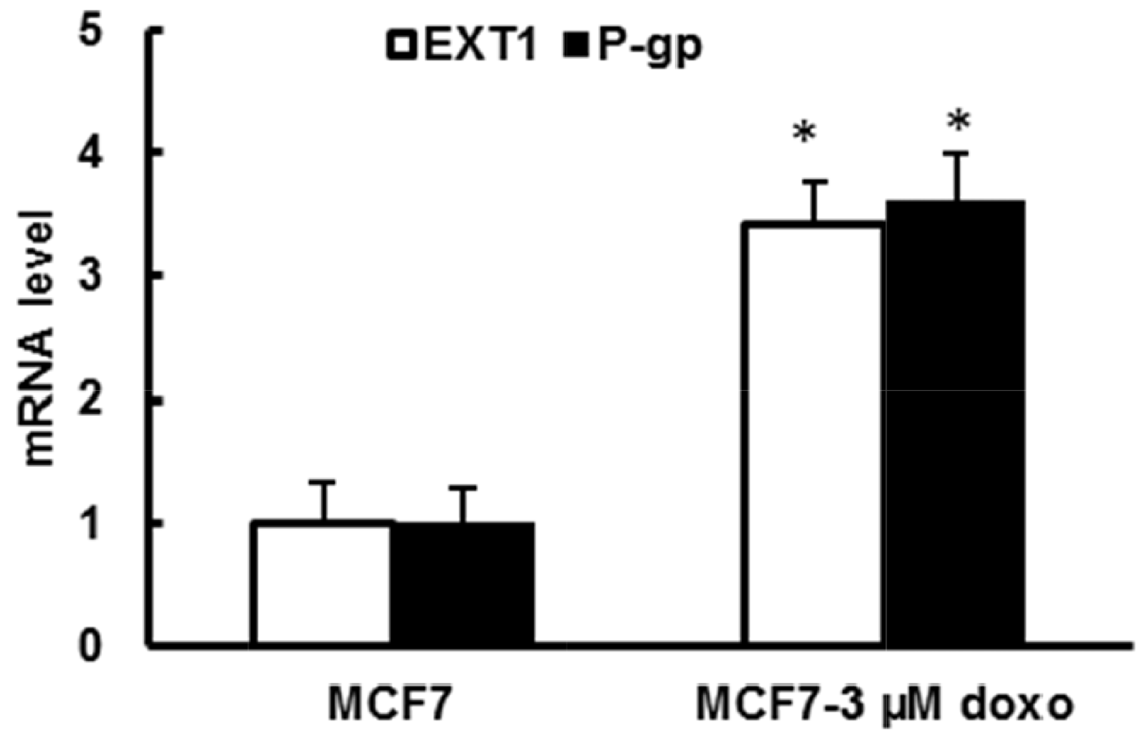

Supplemenatry Figure 2: Acute doxo treatment enhances EXT1 mRNA expression in MCF7 cells. MCF7 cells were starved for 12 h and treated with 3 µM of doxo for the next 12 h, total RNA extracted was tested for P-gp and EXT1 mRNA expression levels by qRT-PCR. Error bars represent mean ± SD from 3 independent samples. $*p < 0.05$ compared with MCF7.

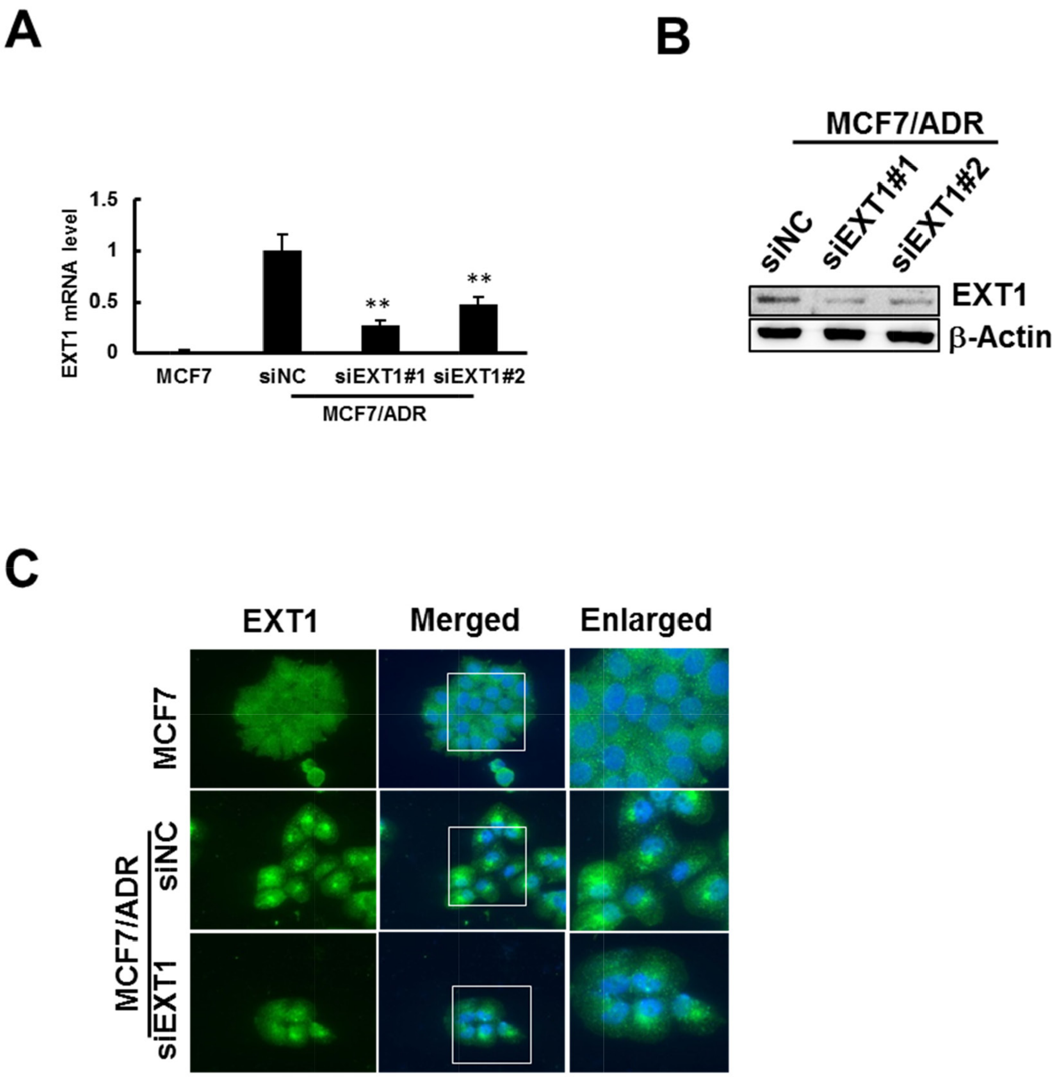

Supplemenatry Figure 3: MCF7/ADR cells express enhanced level of EXT1. MCF7/ADR cells were transfected with 50 nM of negative control siRNA (siNC) or EXT1 targeting siRNA with 2 different constructs (siEXT1#1 and #2) for 48 h. **(A)** EXT1 mRNA level was detected by qRT-PCR. Error bars represent mean ± SD from 3 independent experiments. **$p < 0.01$ compared with MCF7 and **(B)** EXT1 protein was determined by immunoblot analysis. β-actin was used as an internal control. **(C)** EXT1 staining detected with anti-EXT1 antibody after transfection of MCF7/ADR cells with siRNA specific to EXT1 or NC for 48 h.

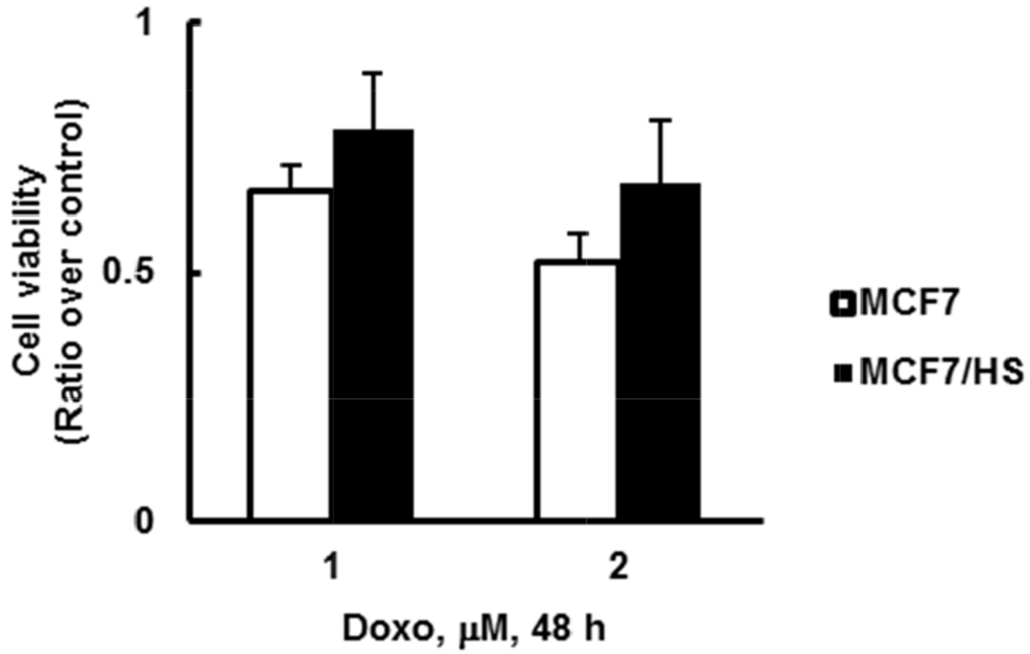

Supplemenatry Figure 4: HS enhances the viability of MCF7 cells challenged with doxo.MCF7 cells were maintained in media containing heparan sulfate for 2–3 weeks and then treated with doxo (1 or 2 µM) for 48 h. MTT assay was performed to compare cell viability among MCF7 maintained in normal media and maintained in heparan sulfate-containing media. Values are means ± SD from 8 sampled wells. Data are expressed as ratio to vehicle control.

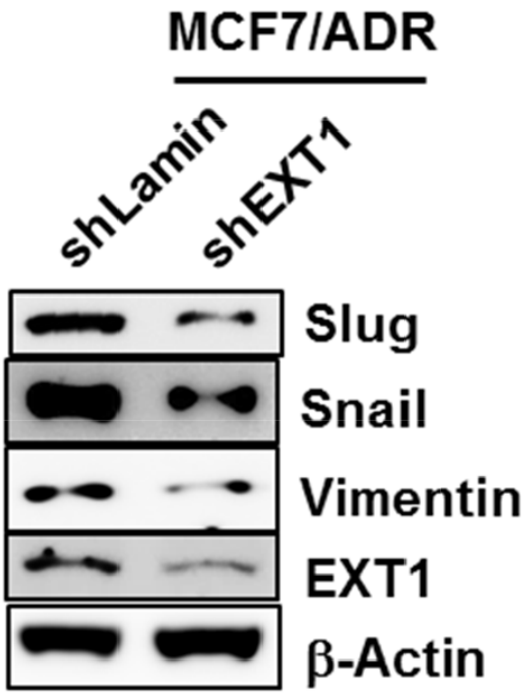

Supplemenatry Figure 5: shRNA mediates stable knockdown of EXT1 and inhibits protein levels for EXT1 and EMT marker genes. Protein levels for EXT1 and EMT marker genes were determined by immunoblot analysis. β-actin used as an internal control.

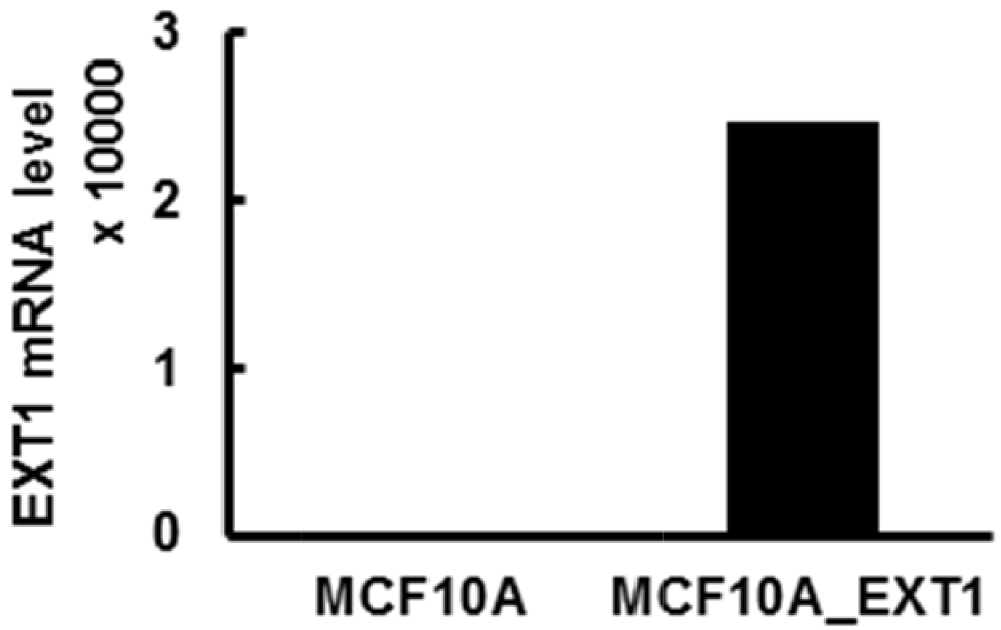

Supplemenatry Figure 6: Transfection of EXT1 plasmid increases EXT1 mRNA in MCF10A. Total RNA extracted was assayed for EXT1 mRNA expression level by qRT-PCR. Error bars represent mean ± SD from 3 independent samples.
